# Aqueous immune mediators in malignant uveal melanomas in comparison to benign pigmented intraocular tumors

**DOI:** 10.1007/s00417-016-3541-5

**Published:** 2016-11-22

**Authors:** Yoshihiko Usui, Kinya Tsubota, Tsuyoshi Agawa, Shunichiro Ueda, Kazuhiko Umazume, Yoko Okunuki, Takeshi Kezuka, Naoyuki Yamakawa, Hiroshi Goto

**Affiliations:** Department of Ophthalmology, Tokyo Medical University Hospital, 6-7-1 Nishi-shinjuku, Shinjuku-ku Tokyo, 160-0023 Japan

**Keywords:** Benign pigmented intraocular tumor, Uveal melanoma, Immune mediator

## Abstract

**Background:**

To examine the usefulness of measuring immune mediators in aqueous humor samples for differentiating malignant uveal melanoma from benign pigmented intraocular tumors.

**Methods:**

Thirteen eyes of 13 patients with uveal melanoma were studied, and 13 eyes of 13 patients with benign pigmented intraocular tumors served as controls. Undiluted samples of aqueous humor were collected, and a cytometric bead array was used to determine the aqueous humor concentrations of 35 immune mediators comprising 14 interleukins (IL), interferon-γ, interferon-γ-inducible protein-10, monocyte chemoattractant protein (MCP)-1, macrophage inflammatory protein (MIP)-1α, MIP-1β, regulated on activation normal T cell expressed and secreted, monokine induced by interferon-γ, basic fibroblast growth factor, Fas ligand, granzyme A, granzyme B, eotaxin, interferon-inducible T-cell alpha chemoattractant, fractalkine, granulocyte macrophage colony-stimulating factor, granulocyte colony-stimulating factor, vascular endothelial growth factor, angiogenin, tumor necrosis factor-α, lymphotoxin-α, and CD40L.

**Results:**

Aqueous humor levels of angiogenin, IL-8, and MCP-1 were significantly higher in eyes with malignant melanoma than in those with benign tumors (*p* < 0.05).

**Conclusions:**

Angiogenin, IL-8, and MCP-1 levels in aqueous humor may be potential markers for distinguishing malignant uveal melanoma from benign pigmented intraocular tumors, and may be a useful adjunct to histomorphology, diagnostic imaging, and other biomarkers for the diagnosis and appropriate clinical management of malignant uveal melanoma.

**Electronic supplementary material:**

The online version of this article (doi:10.1007/s00417-016-3541-5) contains supplementary material, which is available to authorized users.

## Introduction

Differentiation between malignant and benign pigmented intraocular tumors is currently based on comprehensive clinical ophthalmoscopic examination and imaging findings. Histopathological or cytological features are the gold standard for diagnosis of pigmented intraocular tumors, although choroidal biopsy may be inherently associated with some seeding risk. Almost all malignant pigmented intraocular tumors are uveal melanomas. Although many tumor markers for uveal melanoma have been reported, no such marker has been established for differentiating melanoma from benign tumors. Treatment for ocular malignancy is enucleation in certain tumor conditions, and the lack of good diagnostic markers for pigmented uveal tumors may lead to inappropriate or inadequate treatment [1]. Thus, it is important to establish the grade and risk of pigmented intraocular tumors before surgery in order to provide adequate treatment. To this end, new markers that can distinguish malignant uveal melanoma from other benign pigmented tumors are needed. Immune mediators are present in the aqueous humor of eyes with malignant uveal melanoma [2, 3], both as inflammatory reactions and induced by growth of the melanoma. The number of inflammatory cells has been shown to correlate with prognosis [4]. In addition, aqueous flare is influenced by the size of the uveal melanoma [5]. The aim of the present study was to examine the usefulness of measuring immune mediators in aqueous humor samples to differentiate between malignant uveal melanoma and benign pigmented intraocular tumors.

## Methods

**Fig. 1:**
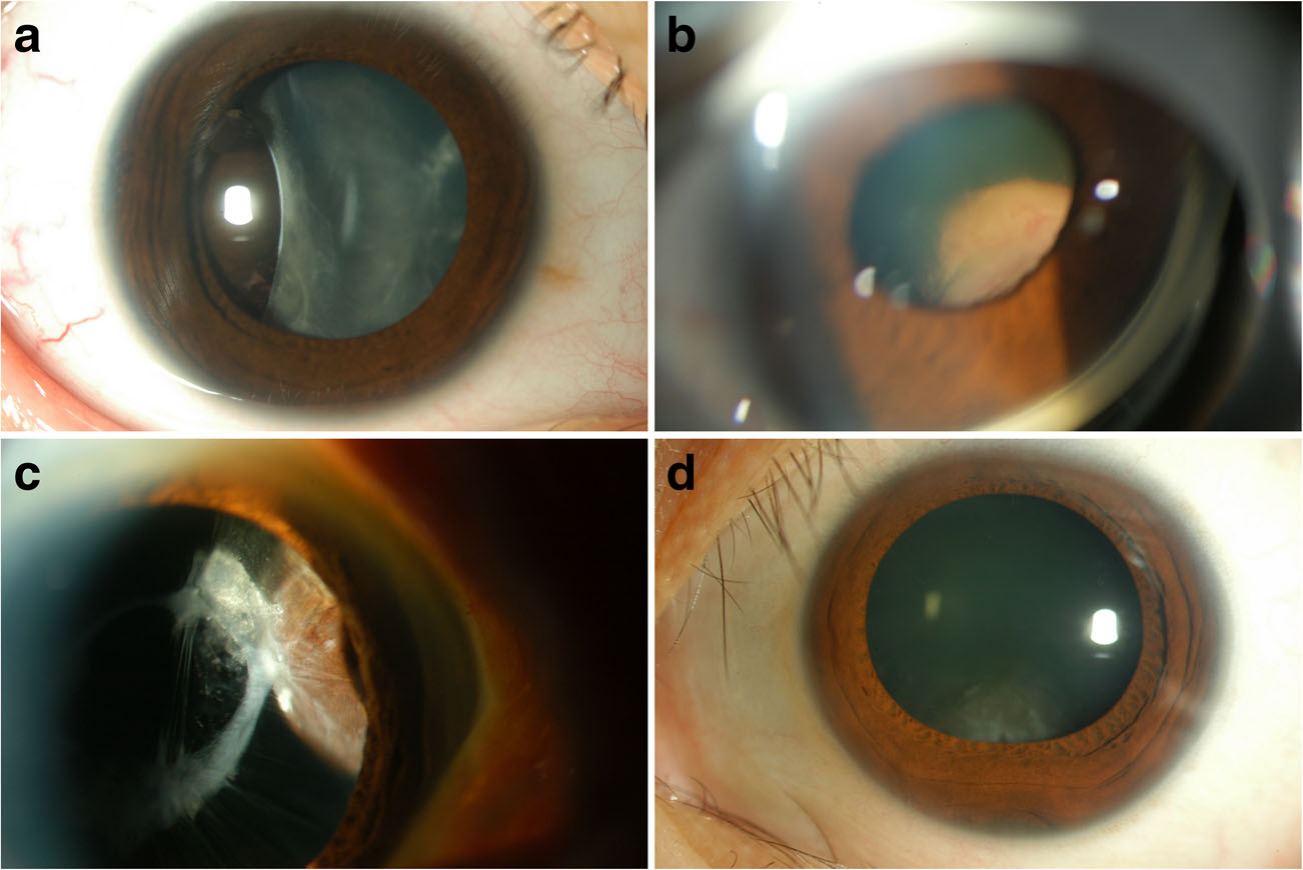
Clinical spectrum of benign pigmented intraocular tumors mimicking malignant uveal melanoma. **a** Case 1: Primary perivascular epithelioid cell tumor (PEComa). Slit-lamp photograph shows a brown mass located temporally, with deformed and tilted lens. **b** Case 2: Mesectodermal leiomyoma. Gonioscopic photograph shows a mass located in the superior nasal quadrant of the ciliary body, displacing the iris and lens. **c** Case 3: Melanocytoma. Slit-lamp photograph shows a pigmented mass located in the nasal quadrant of the ciliary body. **d** Case 4: Melanocytoma. Slit-lamp photograph shows a tumor invading the angle encroaching upon the lens inferiorly

The study included 13 immunocompetent patients (6 men and 7 women; mean age 59.2 ± 14.3 years) with uveal melanoma in the choroid. The control group comprised 13 patients (4 men and 9 women; mean age 57.5 ± 19.1 years), as follows: one patient with a perivascular epithelioid cell tumor in the ciliary body, one with mesectodermal leiomyoma in the ciliary body, four with melanocytoma in the ciliary body, two with adenomas in the ciliary body, three with nevi in the ciliary body, one with nevus in the iris, and one with hypertrophy of the retinal pigment epithelium. Figure 1 shows representative anterior segment photographs of control patients, with findings mimicking malignant uveal melanoma. In all patients, diagnoses were made based on clinical, diagnostic imaging [magnetic resonance imaging (MRI) and B-scan ultrasonography], and histologic data.

Benign pigmented intraocular tumors were treated conservatively in five cases and by local resection in eight cases. Uveal melanomas were treated by enucleation in 12 cases and local resection in one case. Local resection of uveal melanomas was performed as described previously [6]. Briefly, phacoemulsification and aspiration were performed, followed by scleral flap dissection. The base of the sclera and the uvea containing the tumor were incised and subsequently removed, together with the iris, which was radially dissected in advance. After the scleral flap was replaced and sutured, vitrectomy was performed to remove the vitreous hemorrhage.

Undiluted aqueous humor samples were collected before cataract surgery, local resection, or enucleation. The samples were stored immediately at −80 °C until assayed. For histological examination, enucleated eyes or specimens were fixed in 4 % formaldehyde solution and embedded in paraffin. Sections were cut, stained with hematoxylin and eosin, and immunolabeled with human melanoma black 45 (HMB-45) and S100.

The CBA Flex immunoassay kit (BD Biosciences, San Jose, CA, USA) was used to determine the aqueous humor concentrations of 35 immune mediators comprising interleukins (IL)-1α, IL-1β, IL-2, IL-3, IL-4, IL-5, IL-6, IL-8, IL-9, IL-10, IL-11, IL-12p70, IL-17A, and IL-21, interferon (IFN)-γ, interferon-γ-inducible protein (IP)-10, monocyte chemoattractant protein (MCP)-1, macrophage inflammatory protein (MIP)-1α, MIP-1β, regulated on activation normal T cell expressed and secreted (RANTES), monokine induced by interferon-γ (Mig), basic fibroblast growth factor (bFGF), Fas ligand, granzyme A, granzyme B, eotaxin, interferon-inducible T-cell alphachemoattractant (ITAC), fractalkine, granulocyte macrophage colony-stimulating factor (GM-CSF), granulocyte colony-stimulating factor (G-CSF), vascular endothelial growth factor (VEGF), angiogenin, tumor necrosis factor (TNF)-α, lymphotoxin-α, and CD40L. Simultaneous detection of many analytes was able to be performed with a very small sample volume, as described previously [7]. The lowest detectable concentration of this assay was 1.0 pg/ml for all immune mediators tested. This study was approved by the institutional review board of Tokyo Medical University, and all patients gave written informed consent. All patients were Japanese adults.

Statistical analyses were performed using JMP version 12 software (SAS Institute Inc., Cary, NC, USA). Two-group comparisons of numerical variables were performed using Student’s *t* test or the Mann–Whitney *U* test, based on the data distribution pattern. The nonparametric Mann–Whitney *U* test was used to analyze immune mediator levels, because the data were not normally distributed.

## Results

The characteristics of all patients are summarized in Tables 1 and 2, and the aqueous humor levels of the immune mediators are summarized in Table 3. Aqueous humor levels [mean (range) pg/ml] of angiogenin, IL-8, and MCP-1 were significantly higher in uveal melanoma than in benign pigmented intraocular tumors [angiogenin: 21962.4 (4978.7–84479.6) pg/ml vs. 7071.6 (0–11589.6) pg/ml, *p* < 0.05; IL-8: 525.6 (16.0–3428.7) pg/ml vs. 33.3 (0–128.6) pg/ml, *p* < 0.01; MCP-1: 2416.7 (600.1–7723.0) pg/ml vs. 533.7 (117.3–1712.3) pg/ml, *p* < 0.001]. The levels of these three up-regulated immune mediators in individual patients are shown in Tables 1 and 2. The levels of the other immune mediators were not significantly different between the groups, and many were below the level of detection (Table 3).

**Table 1:** Clinical data and aqueous humor levels of angiogenin, IL-8, and MCP-1 in all patients with benign pigmented intraocular tumors

No.	Gender	Age (years)	Size (L, W, H mm)	Tumor location	Histological diagnosis	Presence of subretinal detachment	Angiogenin (pg/ml)	IL-8 (pg/ml)	MCP-1 (pg/ml)	Treatment	Follow-up
1	F	13	10, 9, 8	CB	PEComa	−	5490	5	117	Local resection	71 M
2	F	41	6, 4, 2	CB	Mesectodermal leiomyoma	−	6513	64	332	Local resection	60 M
3	F	46	8, 8, 7	CB	Melanocytoma	−	4244	0	262	Local resection	55 M
4	F	67	10, 9, 7	CB	Melanocytoma	−	8866	129	1712	Local resection	67 M
5	M	59	8, 7, 7	CB	Melanocytoma	+	0	52	1021	Local resection	28 M
6	M	60	6, 5, 5	CB	Melanocytoma	+	5946	7	401	Local resection	64 M
7	F	49	8, 7, 6	CB	Adenoma	−	9616	72	497	Local resection	39 M
8	M	40	6, 4, 6	CB	Adenoma	+	8468	87	544	Local resection	33 M
9	F	70	3, 2, 1	Retina	Hypertrophy of RPE	−	11589	3	514	Observation	27 M
10	M	70	10, 7, 8	Iris	Nevus	−	9592	8	648	Observation	20 M
11	F	78	5, 4, 4	Choroid	Nevus	−	8575	0	328	Observation	69 M
12	F	76	4, 3, 3	Choroid	Nevus	−	4453	7	235	Observation	56 M
13	F	79	3, 2, 1	Choroid	Nevus	−	8575	0	328	Observation	32 M

IL = interleukin; MCP = monocyte chemoattractant protein; F = female; M = male; NA = not available; CB = ciliary body; PEComa = primary perivascular epithelioid cell tumor; RPE = retinal pigment epithelium

**Table 2:** Clinical data and aqueous humor levels of angiogenin, IL-8, and MCP-1 in all patients with uveal melanoma

No.	Gender	Age (years)	Size (L, W, H mm)	Tumor location	Histological type	Presence of subretinal detachment	Angiogenin (pg/ml)	IL-8 (pg/ml)	MCP-1 (pg/ml)	Treatment
1	F	75	16, 12, 7	Choroid	Spindle B	−	9022	287	1198	Enucleation
2	F	85	8, 2, 2	Choroid	Mixed	−	5432	34	600	Local resection
3	M	59	18, 12, 10	Choroid	Mixed	+	13446	17	636	Enucleation
4	M	56	3, 3, 1	Choroid	Mixed	−	16521	76	1805	Enucleation
5	F	65	9, 8, 8	Choroid	Mixed	+	18920	63	2683	Enucleation
6	F	57	9, 8, 6	Choroid	Mixed	−	14480	419	3220	Enucleation
7	F	37	15, 13, 12	Choroid	Mixed	−-	6824	3429	7723	Enucleation
8	M	54	14, 6, 7	Choroid	Mixed	+	4979	2153	7277	Enucleation
9	F	41	12, 11, 10	Choroid	Mixed	+	5252	16	910	Enucleation
10	M	41	15, 12, 9	Choroid	Mixed	+	23717	114	1911	Enucleation
11	F	76	16, 11, 14	Choroid	Mixed	+	70877	100	1511	Enucleation
12	M	59	14, 14, 12	Choroid	Mixed	+	11162	55	721	Enucleation
13	M	65	16, 14, 12	Choroid	Mixed	+	84480	68	1222	Enucleation

IL = interleukin; MCP = monocyte chemoattractant protein; F = female; M = male; NA = not available

**Table 3:** Immune mediator levels in aqueous humor of patients with malignant and benign pigmented intraocular tumors

	Malignant (*n* = 13)	Benign (*n* = 13)	*p* value
Angiogenin (pg/ml)	21962.4 (4978.7−84479.6)	7071.6 (0−11589.6)	0.0257
bFGF (pg/ml)	21.0 (0−259.7)	6.1 (0−27.7)	0.6629
CD40 ligand (pg/ml)	0	0	
Eotaxin (pg/ml)	0	0	
Fas ligand (pg/ml)	75.9 (0−807.7)	157.2 (0−1565.4)	0.3833
Fractalkine (pg/ml)	3.2 (0−15.9)	23.8 (0−298.8)	0.6081
G-CSF (pg/ml)	1.1 (0−7.8)	14.6 (0−105.3)	0.3833
GM-CSF (pg/ml)	0	0	
Granzyme A (pg/ml)	0.3 (0−1.8)	7.7 (0−74.5)	0.5554
Granzyme B (pg/ml)	0.3 (0−3.7)	13.6 (0−167.6)	0.4887
IFN-γ (pg/ml)	0.4 (0−5.4)	0.5 (0−6.3)	0.9795
IL-1α (pg/ml)	0	0	
IL-1β (pg/ml)	0	0	
IL-2 (pg/ml)	0	0	
IL-3 (pg/ml)	0	0	
IL-4 (pg/ml)	0	0	
IL-5 (pg/ml)	2.7 (0−19.2)	0	0.505
IL-6 (pg/ml)	1185.8 (2.1−13635.8)	196.8 (0−683.4)	0.7005
IL-8 (pg/ml)	525.6 (16.0−3428.7)	33.3 (0−128.6)	0.0052
IL-9 (pg/ml)	0	0	
IL-10 (pg/ml)	2.9 (0−27.7)	0	0.3173
IL-11 (pg/ml)	0.8 (0−10.3)	15.0 (0−194.8)	0.9795
IL-12p70 (pg/ml)	0	0	
IL-17A (pg/ml)	0.4 (0−2.6)	5.5 (0−68.7)	0.9183
IL-21 (pg/ml)	3.0 (0−38.6)	41.3 (0−500.5)	0.7389
IP-10 (pg/ml)	1133.9 (60.8−7540.0)	310.0 (0−1550.4)	0.0687
ITAC (pg/ml)	0	0	
LT-α (pg/ml)	0	0	
MCP-1 (pg/ml)	2416.7 (600.1−7723.0)	533.7 (117.3−1712.3)	0.0002
Mig (pg/ml)	1727.8 (0−13496.1)	141.9 (11.8−553.6)	0.209
MIP-1α (pg/ml)	0.2 (0−2.5)	0.9 (0−10.4)	0.7389
MIP-1β (pg/ml)	21.9 (0−125.4)	22.3 (0−64.8)	0.7005
RANTES (pg/ml)	131.5 (0−1710.9)	15.9 (0−207.3)	0.2592
TNF-α (pg/ml)	0	0	
VEGF (pg/ml)	275.0 (0−1993.7)	55.4 (0−146.0)	0.1824

Immune mediator levels are expressed as mean (range).

bFGF = basic fibroblast growth factor; G-CSF = granulocyte colony-stimulating factor;

GM-CSF = granulocyte macrophage colony-stimulating factor; IFN = interferon; IL = interleukin;

IP-10 = interferon gamma-induced protein 10 kDa; ITAC = interferon-inducible T-cell alphachemoattractant;

LT-α = lymphotoxin-α; MCP = monocyte chemoattractant protein;

Mig = monokine induced by interferon γ; MIP = macrophage inflammatory protein;

RANTES = regulated upon activation normal T expressed and presumably secreted;

TNF = tumor necrosis factor; VEGF = vascular endothelial growth factor

Although aqueous VEGF concentration has been reported to be significantly up-regulated in uveal melanoma compared to cataract, the aqueous humor level of VEGF in uveal melanoma in this study was not significantly elevated compared to benign intraocular pigmented tumors [mean (range) VEGF level: 275.0 (0–1993.7) pg/ml vs. 55.4 (0–146.0) pg/ml, *p* = 0.18]. We found no expression or very low levels of VEGF (<50 pg/ml) in 23.1 % of the 13 patients.

Other ocular lesions may mimic melanoma (pseudomelanomas), and age-related macular degeneration (AMD) is one of the pseudomelanomas commonly encountered clinically [8]. Therefore, we compared cytokine expression profiles in uveal melanoma and ARMD. Angiogenin, MCP-1, and IL-8 were significantly up-regulated in uveal melanoma compared to ARMD (Supplementary Table 1), similar to the results of the comparison with benign tumors. In addition, IL-6, Mig, and IP-10 levels were higher in uveal melanoma than in ARMD, probably due to the stronger inflammatory state in uveal melanoma.

## Discussion

Uveal melanoma often causes ocular inflammation and flare in the anterior chamber [5, 9]. Tumor-infiltrating lymphocytes are detected in 5 % to 12 % of uveal melanoma [4], and correlate with high flare values [9]. Infiltrating inflammatory cells such as macrophages and lymphocytes often exist in cancer tissues, and are believed to exert anti-tumor effects through immune surveillance. However, inflammation in the tumor microenvironment has many cancer-promoting effects that facilitate the proliferation and survival of malignant tumors, and promote angiogenesis and metastasis [10]. Various cytokines are detected in uveal melanoma, and they are produced by either tumor cells or infiltrating immune cells [11]. Melanoma cells in culture express large numbers of cytokines and growth factors [12, 13], and melanoma-derived cytokines and growth factors exert paracrine effects on intraocular and inflammatory cells. Conversely, cytokines and growth factors produced by inflammatory tumor cells can stimulate malignant cells. Thus, cytokines and growth factors in the uveal melanoma microenvironment may regulate the functions of both malignant and inflammatory cells, possibly affecting the mechanism of tumor progression.

To the best of our knowledge, there are no reports of markers for differentiating uveal melanoma from benign pigmented intraocular tumors. We previously described the presence of cystoid macular edema due to a high concentration of VEGF in the vitreous [14], which was tentatively explained by the presence and elevated level of VEGF in benign intraocular tumors. Several studies have demonstrated an essential role of VEGF as a prognostic marker of the aggressiveness of uveal melanoma [2, 15, 16]. In the present study, the aqueous humor VEGF level was not significantly increased in uveal melanoma compared to benign intraocular pigmented tumors. The discrepancy in findings between studies may be explained in part by the selection of controls. When we compared the mean aqueous humor VEGF level in the present series of uveal melanoma patients with that in cataract patients (23.1 pg/ml, n = 30), the level was significantly elevated in uveal melanoma patients (data not shown). Vinores et al. [17] reported VEGF expression in 26 % of uveal melanomas, while others have reported that VEGF was absent in uveal melanomas [18, 19]. Therefore, VEGF may not be a specific marker for malignant uveal melanoma because aqueous humor VEGF levels do not differentiate between benign pigmented intraocular tumors and malignant uveal melanoma.

Angiogenin acts as a growth factor for melanocytes. As such, angiogenin is thought by some to represent an autocrine growth factor in malignant melanoma of the skin, and has been reported to predict treatment response in patients with metastatic cutaneous melanoma [20]. The present study, however, is the first to show upregulation of angiogenin in uveal melanoma. Thus, angiogenin may have an important role in melanoma-related angiogenesis and may represent an important target of anti-angiogenic therapy.

MCP-1 and IL-8—a chemokine and cytokine, respectively, with important roles in inflammation—are potent chemotactic factors primarily for macrophages and neutrophilic granulocytes. IL-8 is known to be a potent growth factor for human malignant melanoma [21–23]. In the present study, both MCP-1 and IL-8 were up-regulated in the aqueous humor of patients with uveal melanoma. Whether uveal melanoma cells or the infiltrating immune cells produced these mediators, however, is unknown. Uveal melanoma is considered an immunogenic tumor, because of the identification of numerous tumor-associated antigens. It is possible that because infiltrating immune cells react more strongly to malignant tumors than to benign tumors, they produce more MCP-1 and IL-8 in response to malignant uveal melanoma. In fact, we found a significant correlation between the number of CD3-positive cells/mm^2^ in melanoma specimens and aqueous MCP-1 (R ^2^ = 0.47, *p* = 0.01) as well as IL-8 levels (*R* ^2^ = 0.63, *p* = 0.0011), but not angiogenin level (*R* ^2^ = 0.007, *p* = 0.78) (data not shown). Furthermore, MCP-1 and IL-8 have been reported to be up-regulated in rhegmatogenous retinal detachment [24]. In the current study, the prevalence of subretinal detachment was higher in uveal melanoma than in benign pigmented tumors (Tables 1 and 2). Therefore, the presence of subretinal detachment may contribute to the elevated aqueous MCP-1 and IL-8 levels in patients with uveal melanoma. Additional studies are needed to elucidate the functional role of angiogenin, IL-8, and MCP-1 in uveal melanoma.

The present study has some limitations, including its retrospective nature, the relatively small sample size, and sample selection. In addition, the study design does not al-low examination of the evolution of the aqueous humor immune mediator profile over time. Further studies with a larger number of the patients and longer follow-up are crucial for confirming the roles of angiogenin, MCP-1, and IL-8. Other types of benign pigmented intraocular tumors, such as hemangioma, osteoma, metastatic uveal tumors, and granuloma of the uvea [8], were not examined. Furthermore, we compared malignant melanoma only with benign pigmented tumors in this study. However, some of the choroidal melanomas are amelanotic, and pigmentation may not be the main discriminating factor. Other non-pigmented tumors that masquerade as malignant melanoma must also be studied. Despite these limitations, however, to our knowledge, this is the first study comparing the usefulness of various immune mediators for distinguishing between malignant uveal melanoma and benign pigmented intraocular tumors.

The results of the present study demonstrate that angiogenin, MCP-1, and IL-8 in the aqueous humor were significantly up-regulated in malignant uveal melanoma and may be potential markers for distinguishing uveal melanoma from benign pigmented intraocular tumors. These markers may also be a useful adjunct to histomorphology and diagnostic imaging techniques such as MRI, optical coherence tomography (OCT), and fluorescein angiography for determining the diagnosis and deciding appropriate clinical management. Large-scale studies are necessary to evaluate whether angiogenin, MCP-1, and IL-8 in the aqueous humor are specific and sensitive markers of malignant uveal melanoma.

## Electronic supplementary material

Below is the link to the electronic supplementary material.

Supplementary Table 1 (PPTX 69 kb)
