# Supplementary material for: Aqueous immune mediators in malignant uveal melanomas in comparison to benign pigmented intraocular tumors
Source: Graefes Arch Clin Exp Ophthalmol. 2016 Nov 22;255(2):393–9. doi: 10.1007/s00417-016-3541-5 (PMC5285432; doi:10.1007/s00417-016-3541-5)
Supplement: Supplementary file 1 — (PPTX 69 kb) [file 417_2016_3541_MOESM1_ESM.pptx]

## Slide 1
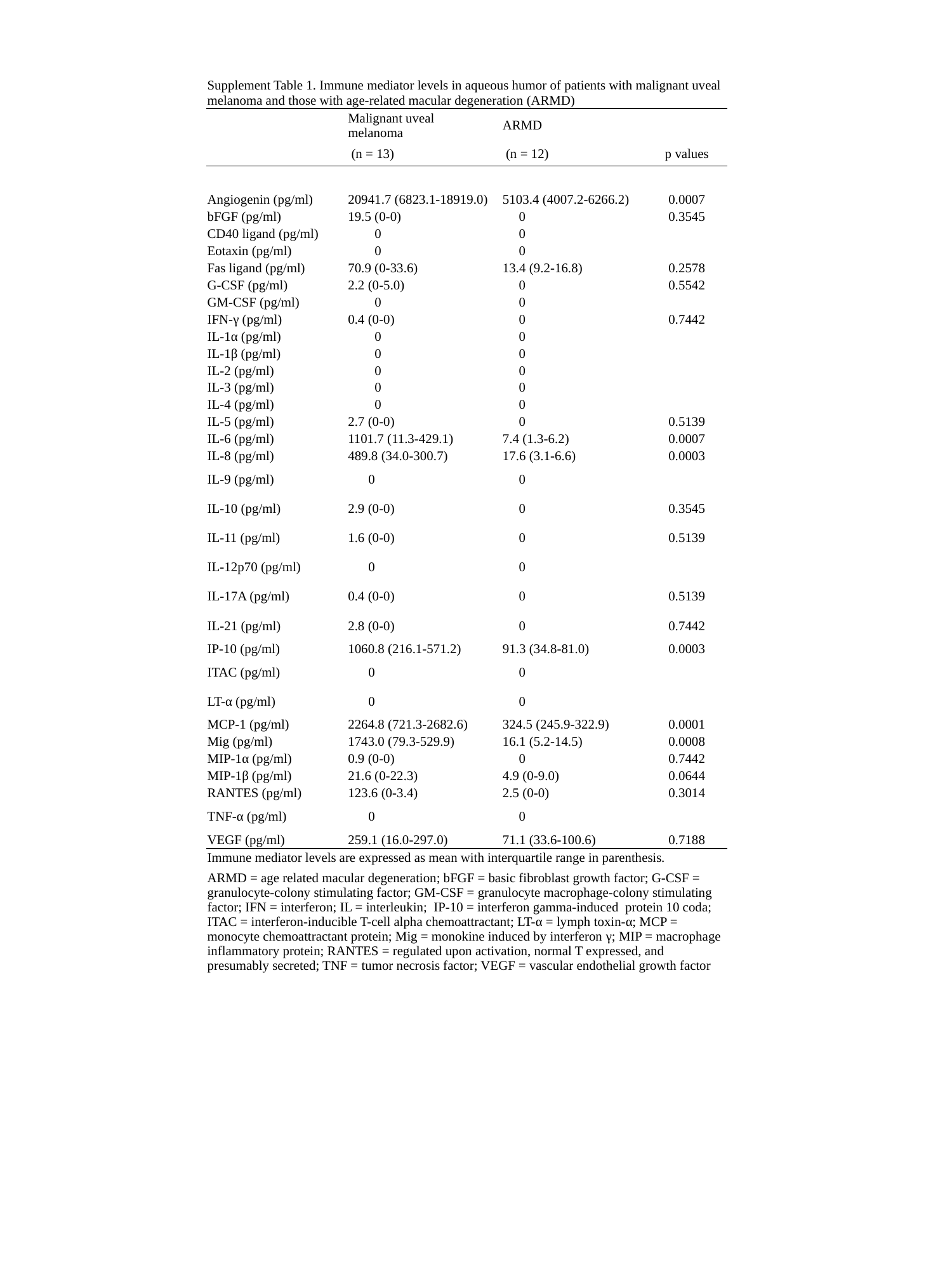

| Supplement Table 1. Immune mediator levels in aqueous humor of patients with malignant uveal melanoma and those with age-related macular degeneration (ARMD) | | | | | | | | | |
| --- | --- | --- | --- | --- | --- | --- | --- | --- | --- |
| | | Malignant uveal melanoma | | | | ARMD | | | |
| | | (n = 13) | | | | (n = 12) | | | p values |
| | | | | | | | | | |
| Angiogenin (pg/ml) | | 20941.7 (6823.1-18919.0) | | | | 5103.4 (4007.2-6266.2) | | | 0.0007 |
| bFGF (pg/ml) | | 19.5 (0-0) | | | | 0 | | | 0.3545 |
| CD40 ligand (pg/ml) | | 0 | | | | 0 | | | |
| Eotaxin (pg/ml) | | 0 | | | | 0 | | | |
| Fas ligand (pg/ml) | | 70.9 (0-33.6) | | | | 13.4 (9.2-16.8) | | | 0.2578 |
| G-CSF (pg/ml) | | 2.2 (0-5.0) | | | | 0 | | | 0.5542 |
| GM-CSF (pg/ml) | | 0 | | | | 0 | | | |
| IFN-γ (pg/ml) | | 0.4 (0-0) | | | | 0 | | | 0.7442 |
| IL-1α (pg/ml) | | 0 | | | | 0 | | | |
| IL-1β (pg/ml) | | 0 | | | | 0 | | | |
| IL-2 (pg/ml) | | 0 | | | | 0 | | | |
| IL-3 (pg/ml) | | 0 | | | | 0 | | | |
| IL-4 (pg/ml) | | 0 | | | | 0 | | | |
| IL-5 (pg/ml) | | 2.7 (0-0) | | | | 0 | | | 0.5139 |
| IL-6 (pg/ml) | | 1101.7 (11.3-429.1) | | | | 7.4 (1.3-6.2) | | | 0.0007 |
| IL-8 (pg/ml) | | 489.8 (34.0-300.7) | | | | 17.6 (3.1-6.6) | | | 0.0003 |
| IL-9 (pg/ml) | | 0 | | | | 0 | | | |
| IL-10 (pg/ml) | | 2.9 (0-0) | | | | 0 | | | 0.3545 |
| IL-11 (pg/ml) | | 1.6 (0-0) | | | | 0 | | | 0.5139 |
| IL-12p70 (pg/ml) | | 0 | | | | 0 | | | |
| IL-17A (pg/ml) | | 0.4 (0-0) | | | | 0 | | | 0.5139 |
| IL-21 (pg/ml) | | 2.8 (0-0) | | | | 0 | | | 0.7442 |
| IP-10 (pg/ml) | | 1060.8 (216.1-571.2) | | | | 91.3 (34.8-81.0) | | | 0.0003 |
| ITAC (pg/ml) | | 0 | | | | 0 | | | |
| LT-α (pg/ml) | | 0 | | | | 0 | | | |
| MCP-1 (pg/ml) | | 2264.8 (721.3-2682.6) | | | | 324.5 (245.9-322.9) | | | 0.0001 |
| Mig (pg/ml) | | 1743.0 (79.3-529.9) | | | | 16.1 (5.2-14.5) | | | 0.0008 |
| MIP-1α (pg/ml) | | 0.9 (0-0) | | | | 0 | | | 0.7442 |
| MIP-1β (pg/ml) | | 21.6 (0-22.3) | | | | 4.9 (0-9.0) | | | 0.0644 |
| RANTES (pg/ml) | | 123.6 (0-3.4) | | | | 2.5 (0-0) | | | 0.3014 |
| TNF-α (pg/ml) | | 0 | | | | 0 | | | |
| VEGF (pg/ml) | | 259.1 (16.0-297.0) | | | | 71.1 (33.6-100.6) | | | 0.7188 |
| Immune mediator levels are expressed as mean with interquartile range in parenthesis. | | | | | | | | | |
| ARMD = age related macular degeneration; bFGF = basic fibroblast growth factor; G-CSF = granulocyte-colony stimulating factor; GM-CSF = granulocyte macrophage-colony stimulating factor; IFN = interferon; IL = interleukin; IP-10 = interferon gamma-induced protein 10 coda; ITAC = interferon-inducible T-cell alpha chemoattractant; LT-α = lymph toxin-α; MCP = monocyte chemoattractant protein; Mig = monokine induced by interferon γ; MIP = macrophage inflammatory protein; RANTES = regulated upon activation, normal T expressed, and presumably secreted; TNF = tumor necrosis factor; VEGF = vascular endothelial growth factor | | | | | | | | | |
